# Co-Transformation of Digital Health and eSport in Metaverse: Moderating Effects of Digital Personality on Mental Health in Multiplayer Online Battle Arena (MOBA)

**DOI:** 10.3390/ijerph20010760

**Published:** 2022-12-31

**Authors:** Lin Cai, Zengsong Huang, Qiujin Feng, Xiaoming Chang, Kexin Yan

**Affiliations:** 1 College of Sport Science, Harbin Normal University, Harbin 150025, China; 2 School of Physical Education, Jiaying University, Meizhou 514015, China; 3 Department of Physical Education, Kunsan National University, 558 Daehakro, Gunsan 54150, Republic of Korea

**Keywords:** mental health, digital personality, eSport performance, metaverse, digital healthcare, theory of behaviourism, cognitive theory, multiplayer online battle arena (MOBA)

## Abstract

Mental health issues (e.g., social exclusion, depression, anxiety, and burnout) became highly prevalent in the global eSport industry. Likewise, the eSport trend in China also dramatically increased, while the attitudes and behaviours of the players also impacted their intentions to utilize video gaming. As China became the epicentre of the online video gaming industry, especially MOBA, it primarily influenced young athletes to adopt video gaming strategies for training purposes. Still, preventive measures are needed for video gaming addictions by athletes to improve their overall eSport performance. To conduct this study, self-administered questionnaires were distributed to 400 athletes aged 18–27 years; the response rate was adequate after screening, out of which 345 were finalized for the data analysis. The results indicate that metaverse-based digital healthcare significantly impacts eSport performance. Moreover, mental health significantly mediated the relationship between metaverse-based digital health and eSport performance. In addition, the digital personality also significantly moderated the relationship between metaverse-based digital healthcare and eSport performance. This research holds tremendous significance both from theoretical and practical perspectives. The study adds valuable insights to the growing body of literature regarding eSport gaming and mental health. The beneficial and constructive intuitions regarding eSport from a psychological perspective can be gained from this study, along with its pros and cons on the mental health of young Chinese athletes.

## 1. Introduction

China possesses one of the largest eSport markets in the world, with a generated revenue of USD 403.1 million in 2021 [1]. Moreover, in the last three decades, eSport developed as one of the multi-million-dollar industries worldwide, specifically the multiplayer online battle arena (MOBA). Here, MOBA is defined as an “online real-time strategy video game”, including the competition between two teams on an already defined battlefield. According to Yang et al. [2], easy accessibility of internet connections in homes digitalized society and helped improve the growth of many economic sectors, including the video game industry. As a result, eSport emerged as one of the favorite leisure activities, especially among young players and athletes worldwide [3]. With the continuous spread of virtual video gaming in China, millions of people are indulging in MOBA, and the number of users is persistently increasing. Although in eSport, the physical activity is low, it still shares some similarities with traditional athletic sports, such as it also includes players in teams or as individuals to compete with the opponents and various matches are held [4]. Various genres are supported by eSport for providing different options to the players. This was also beneficial for the athletes in China to practice and develop required skills via MOBA games before entering into the real-world game field. 

According to Khder et al. [5], gaming became an essential part of humanity. Studies [6,7] also stated that MOBA’s gameplay is quite complicated and dynamic; as a result, athletes experience higher competition levels than in other video games. This often leads to higher frustration among the athletes, making them impatient and tense [8]. Therefore, in China, the I.T. sector is also contributing to the growth of the eSport industry. It also encouraged various Chinese schools, colleges, and sports institutions to utilize MOBA for training purposes. This increased the number of time athletes put into MOBA, resulting in various symptoms, including behavioural salience, lenience, and mood modifications [9]. These symptoms are usually observed in drug addicts. Video game addiction became quite common among young athletes in China, which might impact their overall mental capabilities, resulting in mental exhaustion, frustration, and stress. These adverse outcomes of video gaming also impact the overall eSport performance of the athletes. Pachipala et al. [10] also advocated the negative influences of video gaming addiction on the physical activities of the players. In this regard, various metaverse-based digital healthcare activities are essential for improving the overall eSport performance of the players by providing better healthcare opportunities [11]. The attitudes and behaviours of the players also impact their intentions to utilize video gaming. As China became the epicentre of video gaming, especially MOBA, it primarily influenced young athletes in China to adopt video gaming strategies for training purposes. Still, preventive measures need to be taken for video gaming addictions by athletes to improve their overall eSport performance. 

The video game culture is rapidly booming worldwide. It also involves many professional gamers and athletes from around the world. By the end of 2010, eSport became one of the essential components of the video gaming industry. In this context, various genres of video games were also introduced over the years, which include “multiplayer online battle arena (MOBA), first-person shooter (FPS), fighting, card, battle royale and real-time strategy (RTS) games”. Later, MOBA emerged as one of the essential eSport industry genres, and many athletes also utilize it. However, in 2020, a revenue greater than USD 1 billion was generated by the eSport industry globally, and China contributed to about 35% of the total generated revenue. The easy accessibility of online streaming platforms, such as Twitch and Youtube, also helped promote eSport. Effective use of eSport is considered beneficial for developing specific skills of critical thinking and competitiveness among the players.

In contrast, addiction to video gaming might also lead to harmful addictions. Society plays an essential role in this regard, as it influences the behaviours and attitudes of the players, impacting their overall eSport performance. Additionally, the individuals engaged in eSport are more likely to suffer from physical and mental health conditions [12]. However, no compelling empirical evidence was obtained to determine the impact of mental health on the eSport performance of the players [13,14]. Another limitation observed in past studies [15,16] was that no focus was given on the role of digital personality in the context of eSport. This prevented a better understanding of various internal and external factors on the eSport performance of the players. Therefore, MOBA games, for instance, “League of Legends”, gained more popularity in the past few years due to their complex and dynamic nature, but only a few past studies [17,18] focused on this genre of eSport. As a result, limited literature was attained in the context of MOBA for this study. Thus, the present study effectively determines the impact of metaverse-based digital healthcare on the eSport performance of young Chinese athletes. 

Although the prolonged practice of eSport is considered to impact the mental health of the player, few studies [13,14] also contributed towards the contribution of eSport to the physical health of the players, as the consistent usage of eSport led to headaches and joint pain of the targeted audience. Thus, the present study effectively fills these research gaps observed in previous studies. The novelty is added to the current study by determining the moderating role of digital personality in the context of metaverse-based digital healthcare and the mental health of young Chinese athletes. Based on these research gaps, the following objectives are formulated for the present study to broaden the research area: (a) to explore the impact of metaverse-based digital healthcare on the eSport performance of young Chinese athletes; (b) to determine the mediating role of mental health in the relationship between metaverse-based digital healthcare and eSport performance of young Chinese athletes, and (c) to study the moderating role of digital personality in the relationship between metaverse-based digital healthcare and the mental health of young Chinese athletes. 

The present study provides an adequate theoretical framework for determining the relationship between the constructs under study. This could effectively improve the in-depth knowledge of the role of digital personality and mental health in the relationship between metaverse-based digital health and eSport performance among young athletes in China. This study also promotes various theoretical and practical implications in the eSport industry for ensuring its continuous betterment and growth. This study could also encourage more athletes to adopt MOBA for effective outcomes. Although MOBA games are complicated, they are efficient in developing critical thinking and planning skills among the players. However, along with all the positive impacts of eSport, its negative impacts should also be considered for practical application.

## 2. Theoretical Background and Hypothesis Development

### 2.1. Metaverse-Based Digital Healthcare

Digital healthcare promotes the utilization of technologies to enhance healthcare delivery efficiency. In today’s world, digital healthcare gained a lot of attention for reducing healthcare service costs and preventing various diseases. Metaverse-based digital healthcare integrates healthcare technologies in a virtual setting presenting the real-world scenario [5,6]. It also contributed towards preventing healthcare issues in the context of the eSport industry. According to BAHRİLLİ et al. [19], eSport not only gained attention on social media and other platforms, but also gained the attention of digital healthcare. Although modern technologies helped carry out different routine operations, excessive use also negatively impacts an individual’s health. Past studies [20,21] concluded the positive impact of digital healthcare in dealing with technology anxiety issues and other health-related issues, such as technostress, depression, obesity, and others. It was observed that the usage of computers and other such technologies for a long time not only impacts the user’s mental health, but also negatively impacts the user’s physical health, resulting in joint pains, headaches, and other such issues [22]. Therefore, accurate information and health-related guidelines need to be provided to the users to prevent such issues. Scholars [23] believe that athletes are more likely to suffer from various mental and physical health conditions due to excessive use of eSport, and digital healthcare is effective in improving these conditions.

### 2.2. Mental Health

Mental health incorporates an individual’s social, emotional, and psychological well-being and primarily impacts every phase of life [24]. Both mental and physical well-being are crucial for the adequate performance of a professional. Various intrinsic and extrinsic factors impact people’s mental health, resulting in poor physical performance. According to Savage and Reilly [25], a positive link exists between a professional’s mental and physical performance, especially in the context of an athlete. Mental health is of great concern. Previous studies [26,27] showed that athletes with stringent training sessions and hectic routines are more likely to suffer from various mental health-related issues that negatively influence their game field performance. Although in today’s world of modern technology, various new and advanced technologies were developed to support various professionals by lowering pressure and encouraging their productivity [28].

Similarly, the application of I.T. in the sports industry promoted the development of eSport, encouraging more athletes to utilize video gaming strategies for training purposes. However, the excessive use of eSport resulted in increased video gaming addiction cases, leading to performance anxiety and technostress. Scholars [29] believe that excessive use of video games in eSport also leads to gaming disorders. Most players utilize eSport as a doorway to escape the difficulties of real life. Luo et al. [1] also supported the positive impacts of eSport, stating that it helps in the player’s self-development and improves motivation and self-efficacy. In contrast, the extrinsic motivation factors of eSport include money and fame. Thus, it was observed that mental health is a crucial aspect of an athlete’s and other professionals’ overall performance.

### 2.3. eSport Performance

eSport was effective in improving the cognitive abilities of the players. However, the promotion of a sedentary lifestyle due to intensive video gaming activities resulted in various skeletal muscle disorders in regular players. To avoid such adverse outcomes, more physical activities are being promoted in the context of eSport. Previous research [30] also advocated the significance of eSport in improving the player’s cognitive abilities. Moreover, according to Toth et al. [31], the involvement of physical activities in eSport also helped improve players’ learning abilities, attention, and memory. Therefore, the implementation of physical activities in eSport is still a common topic of debate among various scholars. No detailed understanding is attained in determining the impact of physical activities on eSport performance. Studies [32,33] also supported the role of eSport in increasing competitiveness among the players, which helps them to utilize their capabilities effectively to achieve the desired goals; eSport also increases players’ motivation and encourages them to perform their best. Considering the positive outcomes of eSport, it is largely being used in training the athletes around the globe for improving their cognitive and planning abilities [7]. MOBA games are effective in this context, as their dynamic nature develops essential analytical skills among the athletes for ensuring success in the real-world game field. These games involve the video games in which the competition between two teams is promoted [33,34]. 

### 2.4. Digital Personality

Digital personality includes an individual’s social roles, interests, attitudes, and other traits regarding their technology usage [34]. Scholars [35] believe that personality traits are crucial in predicting the outcomes in various areas of life, such as physical health, job performance, academic achievement, mental health, and longevity. In the metaverse world, people have the freedom to change their personalities, and advanced technologies are also considered to play an essential role in this process. Previous studies [36,37] stated that individuals with practical technical skills and knowledge are more indulged in using technologies for carrying out various operations; the individuals with less technical knowledge and skills are hesitant towards using modern technologies. Digital personality became one of the main concepts in psychology research, and various studies were performed in this context over the years. One of these studies [38] presented a positive relationship between the digital personality and technological performance of an individual. Therefore, digital personality also emerged as an important factor in context of eSport, as it encourages more athletes and capable players to utilize video gaming strategies for positive outcomes.

### 2.5. Theoretical Framework and Hypothesis Development

According to Peck and Dorricott [39], the rapid advancements in the technological era revolutionized digital healthcare services for people, resulting in the proper provision of convenient resources to deal with all their queries. This leads to reduced healthcare service costs and results in positive consequences for a person. A person’s performance is linked directly to psychological stability and peace of mind [40]. The critical expertise required to accomplish up-to-the-mark performance is the basis for the digital sound stability of an individual. According to cognitive theory, how and what people think leads them towards emotional arousal, and specific thoughts or beliefs result in disturbing emotions and negative behaviours. Contrary to this, the theory supports that positive thinking leads towards adaptive behaviours and healthy conduct [41]. As supported by the theory, eSport performance is effective in enhancing an individual’s cognitive abilities. More indulgence in physical activities enhances an individual’s cognitive abilities and critical thinking factors [42]. Digital healthcare in the virtual environment is improved when a person seems to be involved in critical thinking or being creative. This can be possible through eSport indulgence because regular online gaming helps prohibit the person’s being useless and idleness [43]. Digital healthcare can have a massive effect on the ability of an athlete to enhance his performance and improve with time [44]. According to the study by Duggal and Brindle [45], digital healthcare is improved through challenging exposure to games and increased critical thinking ability. Mathews et al. [46] added that digital health in a virtual environment positively correlates with sport performance improvement because scholars Awad et al. [47] investigated sports athletes probably suffering from different issues regarding physical and mental health. Digital healthcare is thus regarded as a great source of aid for improving health issues [48]. Thus, the following hypothesis is formulated:

**Hypothesis** **1** **(H1).** *Metaverse-based digital healthcare significantly impacts eSport performance*.

According to the studies of Fraser and Garcia [49,50], the balancing and stabilization of mental health are crucial, as it results in an individual’s performance improvement. Ma et al. [44] said that the digital provision of healthcare services will be useless if the mind is not ready to perceive and accept those prescriptions. It means that mental health stability and peace of mind add towards performance enhancement and increment to a greater extent. Ramos and Chavira [51] explained that digital technologies became an addiction to youngsters. No matter what information is provided online, if the mind does not accept it, mental health is not stable and cheerful, and the information can be useless. David et al. [52] agree that mental health ensures the person’s performance criteria. eSport athletes are more into critical thinking and mental perspective than physical games. Because online gaming is more about critical minds and command over technology and creativity than other games, such as cricket, tennis, basketball, etc. [53]. According to the theory of behaviourism, behaviours are replicated and imitated through environmental interaction. The person’s performance is impacted when he interacts with others regularly with a different nature and personality than him [54]; eSport, however, leads the athletes to critically observe phenomenons in a certain way by playing games over and over again. eSports encouraged athletes to utilize video games for training. DiFrancisco-Donoghue and Balentine [55] stated that more use of eSport consequently caused enhanced addiction to these games, and also caused mental problems, such as technostress and performance anxiety [56]. The urge to win and play created a sense of rushing attitude among athletes with an increasing trend of eSport prevalence. Excess addiction leads to gaming disorders. Through a study by Burxonovich [57,58], it was observed that most athletes who are addicted to playing games excessively are disturbed internally, and they found a way to escape from reality. Some studies dictate that eSport positively impacts a person’s mental stability and health and inculcates a person’s progressive attitude and critical thinking development [59,60]. Thus the following hypothesis can be formulated:

**Hypothesis** **2** **(H2).** *Mental health significantly mediates the relationship between metaverse-based digital healthcare and eSport performance*.

Every person owns an online personality that differs from their real personality. Some scholars argue that most people possess the same digital personality in real life [61]. A person’s mental health is impacted significantly by the digital role he is observed to play online. The studies of Marchewka et al. [62] clarified that digital personality, which exhibits an individual’s certain behaviours regarding technology, significantly impacts digital healthcare. The person conscious of using technological mediums to deal with his healthcare problems sometimes becomes crumbly and seeks to know more and more. This is due to his habit of relying excessively on technology regarding everything. The person’s digital personality is thus hindering his peace of mind by disturbing it [63,64]. According to the cognitive theory, the impact of a person’s traits and attributes exerts a substantial effect on his actions, which is the central idea for this topic. A confident attitude or behaviour of a person online or offline impacts his real-life behaviours [65]. The digital personality concept also supports this entire sequence of effects. These individual’s minor details perceived to be confirmed exert an impact on his mental peace of mind.

Any initiatives of an individual are due to their innate traits and personal characteristics. If an individual perceives a particular outcome as positive, he intends to perform such tasks [34,66]. This ensures their peace of mind by contributing towards positive mental health. Athletes with command over technological skills and dominance confidently ensure better performance due to their mental support and indirect peace of mind. Their positive digital performance can increase their chances of success and accomplishment [67,68]. Digital healthcare thus resultantly increases and balances an athlete’s mental health as long as he entails a positive or negative digital personality. The digital personality can mould the whole linkage, resulting in a possible variation in this fundamental connection.

**Figure 1:**
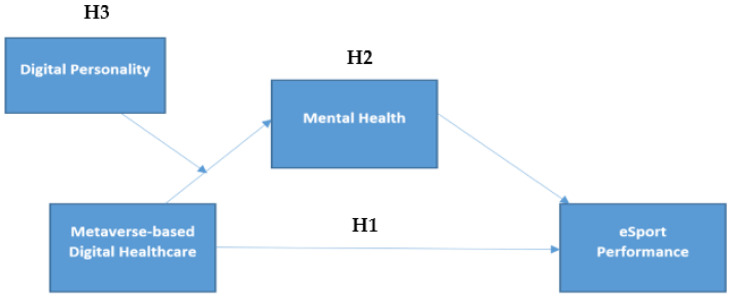
Conceptual model.

The moderated-mediation model of eSport performance as predicted by digital personality, mental health and metaverse-based digital healthcare has been represented as Figure 1.

**Hypothesis** **3** **(H3).** *Digital personality significantly moderates the relationship between metaverse-based digital healthcare and eSport performance*.

## 3. Methods

### 3.1. Research Design, Sampling, and Procedure

The research was conducted following the exploratory research method, and a quantitative data collection technique was used. The aimed research participants for this study are young Chinese athletes aged 18 to 27 years. This sample population ensures the genuine representation of young athletes. The survey-based methodology was incorporated to collect data from the targeted Chinese athletes. Moreover, the appropriateness of the sample must be substantiated to obtain a representative sample.

Furthermore, the sampling technique enables the researchers to formulate judgments regarding a group without considering each individual. Thus, convenience sampling was incorporated into this research to select a representative sample from the entire population. In quintessence, this sampling incorporates information collected from the individuals of the population who were conveniently available and agreed to participate in the research. The questionnaires were formulated based on the self-administration technique have numerous advantages, among which, rapid response and low cost are the most prominent. The data are thus collected through questionnaires in the quantitative studies. The participants must select either “strongly disagree” or “strongly agree” for each of the close-ended items included in the survey. The targeted Chinese athletes were provided access to 400 questionnaires. The information was then collected from four main cities in China, including Shenzhen, Beijing, Guangzhou, and Shanghai. Out of 400 questionnaires distributed, 345 were accurate and complete for this research.

Furthermore, the G* power version 3 software was utilized to corroborate whether the sample size was sufficient or not based on the statistical contemplations. For data analysis, SPSS and SEM-AMOS statistical software were used. This software is also referred to as a casual demonstrating and covariance examination software. AMOS is a technique for graphically modelling structural equations. The most widely used technique in quantitative modelling is structural equation modelling SEM. In addition, it has the potential strength to encounter significant applicable questions. This section elaborates on a detailed data collection process, as the respondents of this research were Chinese athletes. For the selection of the sample in this research, the non-probability sampling technique was incorporated. Convenient sampling was thus utilized to select the sample. It is a data collection technique from the accessible and conveniently available sample. Before data collection and distribution of questionnaires, the consent and complete agreement of the participants were obtained. The questionnaires were distributed to the respondents with a positive response. The targeted participants briefly explained the questionnaires to ensure their acquaintance and clarity regarding the questions asked. For this research, the response rate expected was 50% according to the primary research, so, for the appropriate set of usable data, 400 questionnaires were distributed; out of the distributed questionnaires, 361 were acquired back, and thus the data useable were 345, as indicated in Table 1. The data were collected from the top four metropolitan cities of China (i.e., Beijing, Shanghai, Shenzhen, and Guangzhou).

### 3.2. Measures

The questionnaires were formulated using the five-point Likert scale in this research. It accurately collects data from a vast population, particularly with high-sensitive issues. For the data collection, survey methodology was used. The survey-based methodology is considered appropriate for collecting data from the respondents because it is convenient to disperse and collect the data for analysis. Thus, the research taught the survey-based methodology for collecting data. The decided scales were chosen from additional research, with a careful analysis regarding their reliability and face validity in the previous research. A broad study of the literature with the relevant constructs was held before the establishment of the measuring items. A request to four experts was made to assess the validity of the content of the questionnaires to ensure that it was convenient and easily understandable. The results were then included and added based on the Likert scale ranging from 1 to 5, where one was referred to as strongly disagree, and five meant strongly agree. The option 2 was referred to as disagree, 3 indicated neutral, and 4 referred to agree. For the measurement of metaverse-based digital healthcare, we used a three items scale developed by Tresp et al. [69] (e.g., 1. I know how to use the internet for the health-related questions); the digital personality was measured with the help of a 4-item scale that was supported with good reliability and validity in the past research by Jackson [70] (e.g., I own a different personality virtually). To measure mental health, we relied on a 5-item scale proposed by Baker and Schulberg [71], which also had rugged reliability in the past research (e.g., Did you recently felt to take care of your daily chores?). To measure eSport performance, a 5-item scale [72] was used (e.g., I need a sport experienced trainer that could help me fine-tune my eSport performance).

## 4. Results

### 4.1. Descriptive Statistics

Table 2 explains the summary statistics for the model variables. The minimum and maximum values of the constructs determine that the data were free from outliers, as a range of values projected the threshold imitated by the measurement scale. The mean variables range from 3.2 to 3.4, exhibiting a trend of agreeableness between the participants. Furthermore, as illustrated by the skewness test, the normality values were between −1 and +1, thus determining that all the constructs were distributed normally.

### 4.2. Reliability and Validity Tests

The validity and reliability tests included a series of testing incorporating the KMO and Bartlett test, factor loading, and validity tests through the construct validity. Table 3 explains the results of the KMO and Bartlett tests. The initial step in the validity and reliability testing was the KMO and Bartlett test, which was undertaken to ascertain the sample adequacy. The test was performed to study whether the sample was appropriate, corresponding to the number of variables chosen for the study. The KMO test updates the adequateness of the sample and indicates whether the factor analysis would yield successful results. The Bartlett test is a supplementary test that was implemented to measure the potential association among the variables. In Table 3, the KMO test clutches a value of 0.892, which is greater than 0.5 and contributes to the sample adequacy corresponding to the number of items selected. The Bartlett test is also essential, exhibiting that the variable’s connections among variables would be significant. This result designates that the factor analysis will yield relevant results.

The next test in the reliability and validity testing process is factor analysis. Table 4 determines the factor loading results for the measurement model. The value loading of all the observed variables has a loading between 0.5 and 0.8, which is higher than the threshold limit of 0.5. These results explain that the variables were successfully loaded and strengthened inter-relations. Moreover, no cross-loading was observed among the individual items; thus, exhibiting factor analysis was significant.

The next test, the series of validity testing, was the assessment of the construct validity of the measurement model. The AVE and C.R. were used to study the internal consistency of the constructs through convergent validity. The range of the C.R. values resulting through analysis was between 0.8 and 0.9, which was extensively greater than the threshold-defined range of 0.7. The AVE values of the variables fall between 0.6 and 0.8, more significant than the cut-off value of 0.5. These values implied that the variables were internally steady and, therefore, reliable.

Based on the method proposed by Fornell and Larcker, discriminant validity was studied. Table 5 explains that the square root of AVE figures is smaller than the AVE. Moreover, the inter-construct correlations were smaller than the correlations of intra-variable; thus indicating that the variables were significantly linked with one another, and no other connection demonstrates the linkage more significantly than the intra-variable associations. Therefore, it can be observed that discriminant validity exists in the measurement model. Since the testing series of the KMO and Bartlett test, factor loadings and the construct’s validity all yielded significant results, it is evident that the sample size was appropriate, the inter-relation of variables is significant, and the constructs are valid and reliable.

### 4.3. The Goodness of Fit

**Figure 2:**
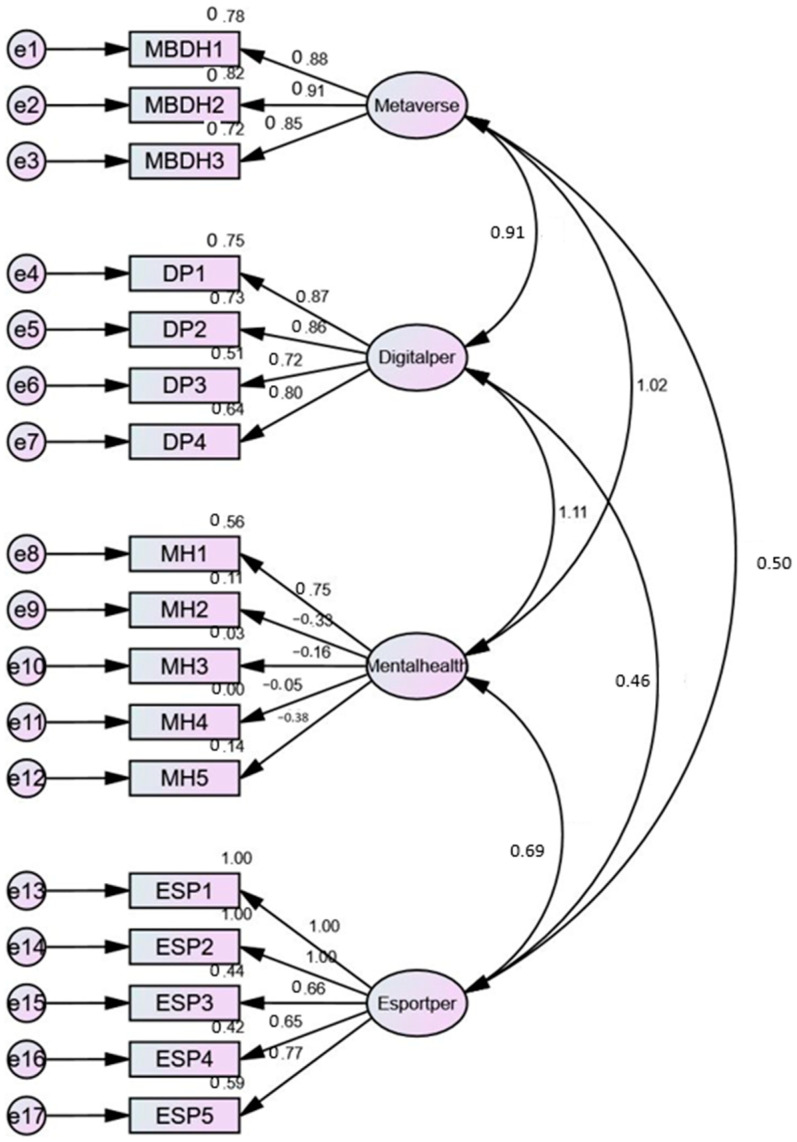
Measurement model.

The model fitness of the measurement model was studied by assessing various fitness indices. The research thus computed CMIN/df, IFI, RMSEA, CFI, and GFI to explore the fitness of the measurement model. Table 6 depicts the computed and threshold figures for every index. It can be observed that the measurement model was completely fit, as all five indices and computed figures relate to the threshold limits. So, the measurement model was fit, and it is supposed that the structural equation modelling would also yield effective results. Figure 2 explains the measurement of the fit model. This picture exhibits the variables’ inter-connectedness level. The variables irrespective of their status (i.e., dependent or independent) are comprised and put together in a diagram to assess their model fitness levels. 

### 4.4. Structural Equation Modelling

**Figure 3:**
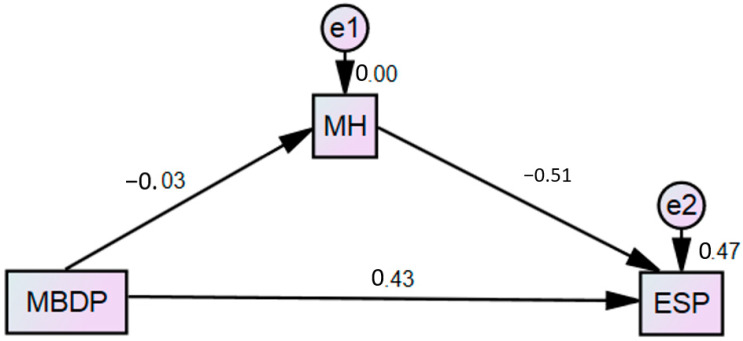
SEM.

**Figure 4:**
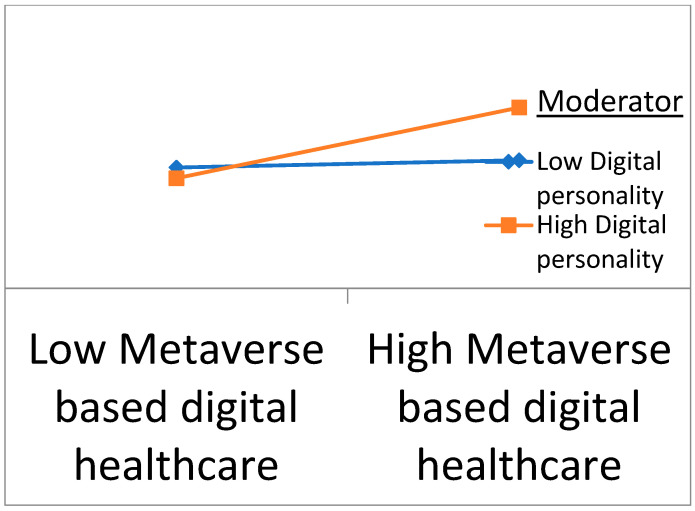
Graphical representation of the moderation hypothesis.

The study hypothesized a significant positive relationship between metaverse-based digital health and eSport performance among Chinese athletes. Table 7 dictates that the linear relationship was significant and positive. A unit change in the metaverse-based digital health would enhance the eSport performance of Chinese athletes by 16.5% (*p* < 0.05). The mediation of mental health between metaverse-based digital health and eSport performance was also significant as the *p*-value, for this formulated hypothesis lies at 0.000. Figure 3 supports the SEM result audacity. The moderation of digital personality between metaverse-based digital health and mental health was also significant, as illustrated in Figure 4 below. 

## 5. Discussion

In the past few years, eSport gained a lot of attention due to its emerging popularity. Various studies focused on the impact of mental health issues in the context of eSport performance, but almost no past study discussed the impact of digital personality in this context. So the present study focused on filling this gap, and the present study’s findings showed that metaverse-based digital healthcare significantly impacts eSport performance. Additionally, mental health also has a significant mediating role in the relationship between metaverse-based digital healthcare and eSport performance, whereas the moderating role of digital personality was also found to be positive in the relationship between metaverse-based digital healthcare and mental health. The present study results show that digital healthcare provides effective services and information to athletes, which helps improve their eSport performance. In this regard, the mental health condition of the athletes also plays an essential role in delivering effective eSport performance. Their digital personalities directly impact the mental health of the athletes. The theoretical framework of these variables is effectively understood by the application of cognitive and behaviourism theories. Both these theories were found to be beneficial in determining the relationships between the constructs of the present study. 

The outcomes of the present study supported the correlation between mental health and the eSport performance of an athlete. The excessive use of eSport is similar to the traditional sport, as it leads to various mental and physical health-related issues. It was observed that athletes playing eSport for a longer time are more likely to suffer from headaches, fatigue, eye pain, and other such issues. Moreover, the involvement of physical activities in eSport is also contributing towards physical injuries, such as back pain, neck ache, wrist pain, and others [73]. Even though various genres of eSport were introduced, one of the most commonly played games is MOBA. These games are beneficial for athletes in developing analytical skills and improving their motivation, but their overuse is also the cause of many health-related issues [3]. Therefore, the mental condition of an athlete impacts his/her overall performance. In this context, the role of digital personality is also crucial, as it includes the behaviours and social roles of the people, which impact their cognitive abilities, affecting their performance. Previous studies [74,75] also advocated the positive role of digital personality in improving the cognitive skills of the players in the context of eSport. Playing electronic games requires a certain set of technical and analytical skills, which improves the self-efficacy of the players encouraging them to perform their best. According to Yılmaz and Özkan [76], efficient healthcare services provide important and accurate information to the players encouraging them to take important measures while playing eSport [15]. This phenomenon is also observed in various sports institutions where various health coaches are also involved in ensuring the effective performance of the athletes by focusing on their mental and physical well-being. Scholars also contributed to the relationship between players’ attitude and behaviour [77,78]. It was observed that the players with high mental capabilities and health perform better than the players with poor psychological health. Thus, in the eSport industry, health-related factors are also considered to be essential for ensuring the effective performance of the athlete.

### 5.1. Theoretical Implications

The present study is effective in improving the in-depth understanding and literature of young Chinese athletes’ eSport performance. This research is one of the pioneers in focusing on the impact of mental health on the eSport performance of Chinese athletes in a metaverse setting. Athletes’ cognitive abilities are also highlighted in this regard, stating the importance of digital personality. The theoretical framework of the present study was developed by utilizing cognitive and behavioural theories. The present study also helped broaden the research areas under these theories. These theories were also effective in explaining the results of the present study. The application of behaviourism theory determines the role of digital personality, and it was stated that the technological attitudes of the players impact their motivation and self-efficacy, leading towards better performance. At the same time, the cognitive theory supported the cognitive mental abilities of the athletes in the context of the positive psychological well-being of the athletes leading towards effective eSport performance. 

This study is also found to be efficient in providing directions for future studies in the context of the eSport industry for providing more empirical evidence. It effectively understood the link between digital healthcare and eSport performance. The negative outcomes of an excessive use of eSport on players’ mental health was also determined in this study, which helped add to the psychological well-being of athletes in the context of eSport. While conducting this study, very little literature was observed in the context of digital personality. This resulted in various difficulties for the researcher. In order to improve the literature in this regard, the moderating role of digital personality in the relationship between metaverse-based digital healthcare and mental health was observed in the present study. It also helped in adding novelty to the current study. As a result of this research, a positive relationship between digital healthcare and eSport performance was observed, which could be an effective source of information for various athletes and their health coaches worldwide. This could also encourage other researchers and scholars to study the significance of mental health in improving the eSport performance of athletes.

### 5.2. Practical Implications

The continuous inclusion of technology in our professional and personal lives largely impacted our mode of action. Similarly, electronic sport or eSport revolutionized the sports industry. Various genres of eSport are being used by various professional players, as well as athletes, for training purposes. The present study helped determine the role of these athletes’ mental health in their eSport performance. This study observed that most of the athletes who were spending most of their time playing eSport suffered from mental exhaustion and eye fatigue. This largely impacted their overall performance. As a result, the health coaches of athletes could also take important measures to ensure the psychological well-being of athletes in this regard. This is also effective in promoting digital healthcare services for positive outcomes. In this regard, the management of various sports companies and agencies could also be appreciated to focus more on the mental health of the athletes to attain better eSport performance. Various mental health-based training sessions could be encouraged to improve the understanding of athletes regarding the significance of mental health for better performance.

The current study is also capable of gaining the attention of the government. It could encourage various private–public partnerships to take initiatives for improving the mental health issues of athletes in the context of eSport. The IT sectors could also be involved in this process, as they would be able to develop software which could be less damaging to the health of the players. Thus, important policies need to be developed in this regard to improve the utilization of eSport for positive outcomes. Excess of anything is considered to be toxic; similarly, the excess usage of eSport is deteriorating the health of athletes and other players. Thus, the current research effectively promotes effective measures to reduce the negative outcomes of excessive eSport usage on people’s mental health by promoting digital healthcare. This ultimately improves the eSport performance of the athletes, improving their motivation. The application of these processes could be effective in the professional career of various athletes worldwide.

### 5.3. Limitations and Future Research

No research study is able to cover a broader research area due to various uncertainties. Similarly, the present study also faces certain limitations due to various reasons. These limitations could be effective for future studies as they could help in paving the way for them. One of the limitations of the present study was the utilization of a single survey method for collecting the data due to a specific timeframe. This prevented the in-depth knowledge of athletes’ experiences while playing eSport. Therefore, future studies could be an opportunity to use more data collection methods, such as interviews and others. As no focus was given to the psychological data of athletes in this study, this gap could also be filled in future studies. The second limitation was the small target audience. Due to easy accessibility and researcher bias, young athletes were targeted for the present study, and no data were collected from elder athletes. This prevented the view of the older generation in the context of eSport. Thus, future studies could be beneficial in providing empirical evidence in this context. The present study was also limited to MOBA games, and data were collected from players of these games, which prevented the discussion of other genres of eSport, such as FPS, RTS, and others. However, future studies could also focus on these genres of eSport for effective comparison and better outcomes. Moreover, in the present study, racial bias was also observed, as only Chinese athletes were considered for collecting the data. This limited the variations in the obtained results, which could be obtained if athletes with different ethnicities and racial backgrounds were considered for data collection. Future researchers could also resolve this issue if efficient measures are taken.

## 6. Conclusions

The main aim of the present study was to determine the influence of digital healthcare on the eSport performance of young Chinese athletes. In this study, the mediating role of mental health was also discussed. Considering the significance of social roles and attitudes towards technology, the moderating role of digital personality was also focused in this regard, adding novelty to the present study. This study was conducted on young Chinese athletes, as they are found to be more indulged in eSport genres. For this purpose, the data were collected from young Chinese athletes playing MOBA games. A survey method was conducted, and about 400 questionnaires were distributed, and only 345 were received for further analysis. Various statistical tools, such as AMOS and SEM, were used to analyze the obtained results. The formulated hypotheses were checked by utilizing the SEM technique, and the current research results show that metaverse-based digital healthcare positively impacts eSport performance. This also showed the importance of psychological well-being for effective performance, and a positive relationship was observed between mental health and eSport performance. As a result, the second formulated hypothesis was also accepted, stating the positive mediating role of mental health in the relationship between metaverse-based digital healthcare and eSport performance. Therefore, the moderating role of digital personality in the context of metaverse-based digital healthcare and the mental health of athletes in MOBA was also found to be significant.

## Figures and Tables

**Table 1:** Survey response rate.

Total distributed questionnaires	400
Received	361
Removing outliers	16
Removal of missing value	345
Usable data finalized	345

**Table 2:** Summary of descriptive statistics.

	N	Minimum	Maximum	Mean	Std. Deviation	Skewness	
	Statistic	Statistic	Statistic	Statistic	Statistic	Statistic	Std. Error
MBDH	345	1.00	5.00	3.2483	1.41147	−0.116	0.201
DP	345	1.00	5.00	3.4983	1.27458	−0.045	0.201
MH	345	1.00	5.00	3.4779	0.74279	0.100	0.201
ESP	345	1.00	5.00	3.2600	0.99320	−0.666	0.201
Valid N (listwise)	345						

Notes: MBDH = metaverse-based digital healthcare, MH = mental health, ESP = eSport performance, and DP = digital performance.

**Table 3:** KMO and Bartlett Test.

Kaiser–Meyer–Olkin Measure of sampling adequacy.		0.892
Bartlett’s test of sphericity	Approx. chi-square	2201.936
	df	136
	Sig.	0.000

**Table 4:** Rotated component matrix.

	1	2	3	4
Metaverse-based digital healthcare				
MBDH1	0.835			
MBDH2	0.820			
MBDH3	0.825			
Digital personality				
DP1		0.864		
DP2		0.866		
DP3		0.702		
DP4		0.785		
Mental health				
MH1			0.720	
MH2			0.609	
MH3			0.925	
MH4			0.921	
MH5			0.606	
eSport performance				
ESP1				0.917
ESP2				0.917
ESP3				0.747
ESP4				0.689
ESP5				0.793

**Table 5:** Convergent and discriminant validity.

	CR	AVE	MSV	MBDH	DP	MH	ESP
MBDH	0.823	0.726	0.352	*0.864*			
DP	0.998	0.675	0.544	0.702	*0.714*		
MH	0.916	0.719	0.598	0.614	0.657	*0.842*	
ESP	0.951	0.827	0.419	0.725	0.586	0.656	*0.898*

Notes: MBDH = metaverse-based digital healthcare, MH = mental health, ESP = eSport performance, and DP = digital performance; the data in the diagonal (in italic) are the square root of AVE of the construct.

**Table 6:** Model fit indices.

CFA Indicators	CMIN/DF	GFI	IF	CFI	RMSEA
Threshold value	≤3	≥0.80	≥0.90	≥0.90	≤0.08
Observed value	2.792	0.831	0.903	0.915	0.078

**Table 7:** Hypothesis Testing Outcomes.

Effects	Hypothesized Path	Β	S.E	*p* Value	Conclusion
Linear effects					
Hypothesis 1 (+)	MBDH → ESP	0.430	0.175	0.001	Accepted
Mediation effect					
Hypothesis 2	MBDH → MH → ESP	0.171	0.271	0.000	Accepted
Moderation effect					
Hypothesis 3	MBDH → DP → MH	0.174	0.168	0.003	Accepted

Notes: MBDH = metaverse-based digital healthcare, MH = mental health, ESP = eSport performance, and DP = digital performance.

## Data Availability

The data are not publicly available due to personal privacy and non-open access to the research program.
